# A Manipulation of Visual Feedback during Gait Training in Parkinson's Disease

**DOI:** 10.1155/2012/508720

**Published:** 2011-09-20

**Authors:** Quincy J. Almeida, Haseel Bhatt

**Affiliations:** Sunlife Financial Movement Disorders Research and Rehabilitation Centre, Wilfrid Laurier University, Waterloo, ON, Canada N2L 3C5

## Abstract

Visual cues are known to improve gait in Parkinson's disease (PD); however, the contribution of optic flow continues to be disputed. This study manipulated transverse line cues during two gait training interventions (6 weeks). PD subjects (*N* = 42) were assigned to one of three groups: treadmill (TG), overground (OG), or control group (CG). Participants walked across lines placed on either treadmills or 16-meter carpets, respectively. The treadmill (TG) offered a reduced dynamic flow from the environment, while lines presented on the ground (OG) emphasized optic flow related to the participant's own displacement. Both interventions significantly improved (and maintained through retention period) step length, thus improving walking velocity. Only the OG improved in the TUG test, while only the TG showed hints of improving (and maintaining) motor symptoms. Since gait improvements were found in both training groups, we conclude that by reducing optic flow, gait benefits associated with visual cueing training can still be achieved.

## 1. Introduction

Individuals with Parkinson's disease (PD) have been shown to walk with a stooped posture, limited arm swing, slow velocity, and small shuffling steps that can often lead to falls [1]. Sensory cueing strategies such as auditory, tactile, and visual cues have often been used to help walking in PD. Stein and Glickstein [2] suggested that of all these modalities, visual cues are most effective in improving PD gait. It is not clear, however, whether improvements might be the result of improved use of optic flow, greater attention directed towards walking, or cortically driven planning of discrete steps that bypass the basal ganglia. 

Optic flow is a prominent theory that is often put forward to explain the benefits associated with using transverse lines. This theory suggests that transverse lines improve walking due to the stripes accentuating the flow of the surrounding environment as one moves through space [3, 4]. This notion of optic flow has been strongly supported by Azulay et al. [5] that believe the lines emphasized optic flow which improved gait velocity and stride length in PD participants. Optic flow has been previously manipulated through either virtual reality or a projected tunnel screen [6, 7], and in each case, manipulation was presented by changing the surrounding environment. An interesting method of manipulating visual information from the surrounding environment is to have people walk on a treadmill. Biomechanically, the differences that exist between treadmill and overground walking are negligible [8]. Interestingly, however, walking on a treadmill allows a reduction of typical optic flow that would normally be associated with every day walking. Song and Hidler [8] and Frankel-Toledo et al. [9] acknowledge that subjects on a treadmill do not receive the same optic flow as they do when walking overground. Bello et al. [10] proposed that gait improvements in PD treadmill walking are caused by the subject's ability to strategically use the distance from the front of the treadmill as a static visual cue. Contrarily, a study by Azulay et al. [5] used stroboscopic lighting to suppress optic flow by transforming stripes on the floor to static cues, resulting rather in a deterioration of gait in PD patients. This contradicting evidence indicates that little is known as to how much, if any, optical flow is needed to improve gait in PD. Thus, comparing overground and treadmill training with identical visual cues provides a unique opportunity to evaluate how optic flow might contribute to gait improvements. 

Fundamental to these gait deficits is the inability to produce a normalized step length [11]. Many popular visually guided cues have been shown to improve step length including the inverted walking stick, projected laser beam [12, 13], and parallel lines [14]. It has been well established that transverse lines an inch wide or more have been best shown to facilitate locomotion [15]. Jiang and Norman [16] found that transverse lines assisted in the initiation of gait in PD individuals. However, most studies that implement transverse lines have often only conducted single sessions [16–18]. Morris et al. [17] showed that a single cueing session was effective in regulating stride length in PD and that a training effect emerged leading to improvements two hours after visual cues were removed. However, the potential for long-term cue training to lead to even longer lasting benefits to gait has yet to be studied. Interestingly, the only case study (with an *n* = 1) using transverse lines as a long-term cueing intervention revealed potential benefits [19]. Thus, more research must explore transverse lines as a long-term cueing therapy for Parkinson's disease. 

Unfortunately, most of the above studies failed to administer a retention assessment; hence, any persisting long-term improvements to gait have yet to be determined. Also, an assessment of gait transference to a more functional test such as the timed up and go (TUG) has not been used, as well as potential symptomatic improvements (UPDRS motor scores). Through the administration of these tests, we can achieve greater insight into the underlying mechanism of improvement with the use of transverse line cues during gait. 

One method of manipulating the provision of transverse lines is to modify the context in which they are provided for training. For example, integrating transverse line cues on a treadmill is novel, as it provides step cues but within a more static background. In contrast, transverse lines provided over the length of a carpet would move past any individual relative to the rest of the surrounding environment. Thus, our study compared two different methods of providing transverse line cues: (1) traditional overground gait training (with transverse lines) and (2) treadmill training. Our primary outcome measure was step length, while additional measures included UPDRS motor scores, lower limb strength gains, TUG times, and other spatiotemporal aspects of gait. All variables were assessed at baseline (pretest), after a 6 week rehabilitation phase (posttest), and 6 weeks later (retention test).

## 2. Methods

### 2.1. Subjects

The study included a total of 42 participants that were assigned to one of three PD groups: treadmill (TG), overground (OG), or control (CG). All participants (recruited through a database from the Sun Life Movement Disorders Research and Rehabilitation Centre, Wilfrid Laurier University, Canada) were diagnosed with Parkinson's disease and then randomized and matched for overall, as well as PD specific demographics (based on a prescreening assessment). 

Each participant tested was confirmed to have clinically typical PD from at least one movement disorders neurologist. All PD patients were responsive to anti-Parkinsonian medication and were in an optimally medicated or “on” medication state at the time of all training and testing sessions. 

Participants were excluded from the study if they had a past history of neurological conditions other than PD or orthopaedic or visual disturbances that severely impaired walking ability. Also, participants were removed if they were unable to independently walk down an 8 meter GAITRite carpet for a total of 10 trials. Each participant was informed of the requirements of the study and signed institutionally approved consent forms, according to the declaration of Helsinki (BMJ 1991; 302: 1194).

### 2.2. Materials

Data was collected in two different rooms, a gymnasium and a laboratory measuring approximately 20 m × 10 m and 9.5 m × 6 m, respectively. Gait data was collected in the gymnasium on a GAITRite carpet (GAITRite, CIR System, Inc., Clifton, NJ, USA) which measured 8 m long × 0.92 m wide and contained sensors that provided footfall information to an attached computer. The 30-second chair stand and TUG test were conducted in the laboratory. Materials needed for the two tests included a straight back chair, a taped line 3 meters away from the chair, and a stop watch. Two Biodex Gait Trainer 2 treadmills were used for the treadmill group, and three 16-meter black landscaping carpets were used for the overground group. Transverse lines were created using white athletic tape.

### 2.3. Protocol

#### 2.3.1. UPDRS Severity Score

All participants' motor symptoms were assessed by a blinded movement disorders specialist using the UPDRS Section 3.

#### 2.3.2. Timed Up and Go (TUG)

TUG test required participants to sit in a chair and when told “go”, participants were asked “to stand up, walk to the taped line, turn around, and sit back in the chair as quickly and safely as possible.” Two trials were performed and time was recorded using a stopwatch. The purpose of the TUG was to assess functional mobility of PD participants and track gait changes over time [20].

#### 2.3.3. 30-Second Chair Stand

The 30-second chair stand required all participants to be seated in a chair and when told “go” to rise to a full stand position and sit back down again. This was repeated as many times as possible in a span of 30 seconds. Two trials were performed, and the total number of stands was recorded. This measure was used to identify any lower limb strength gains that may result from the intervention.

#### 2.3.4. GAITRite Walking

All participants were requested to walk down an 8-meter GAITRite carpet “at a normal casual walking speed” for a total of 5 trials. If participants needed further explanation, they were asked to walk down the carpet as though they were “walking down the street”. Participants started 1 meter before the carpet and told to walk 2 steps beyond the end of the carpet to ensure gait initiation and termination were not processed in data collection. Footfall information was collected to an attached computer, and the following gait measures were obtained: gait velocity (cm/s), cadence (steps/min), mean step length (cm), double support time (s), step time (s), step-to-step variability, step-time variability, and double support variability.

#### 2.3.5. Training Protocol

Participants completed gait training 3 times a week for 6 weeks (18 sessions in total). Each gait session spanned 30 minutes with a mandatory 2-minute break every 8 minutes. However, participants were allowed additional rest if necessary but were required to walk a total of 24 minutes for the gait session to be considered complete. All participants were “on” medication at the time of pre-, post-, and retention testing and during training. All training sessions were conducted at the same scheduled time. Spotters were provided for all participants to ensure safety. In both training groups, visual cues were provided (on ground or treadmill) with the use of white lines (see description below). To standardize the step length required during training, we selected a separation between lines that was a minimum of 8% greater than the initial step length of any of the groups. Thus, based on previous research [12] and also this 8% requirement, the white lines were separated by 70 cm. This ensured that from one consecutive heel strike to the next, participants in both the overground and treadmill group trained with an equivalent distance between cue steps. Furthermore, in order to control for training velocity, stepping was monitored using a timer over the distance covered for the overground group, while velocity could be set manually for the treadmill group. In both cases, training velocity was based on each individuals predetermined self-paced velocity.

**(a) Overground Group**

Overground gait training required participants to walk down equally spaced transverse lines, presented on a 16-meter carpet. The cues were white lines of tape equally distributed at a standardized length on the black background carpet. Participants trained at the same walking speed that was measured at pretest (GAITRite analysis). This was achieved by requiring participants to completely clear the carpet within a specified amount of time. Participants were asked to walk across the lines, turn, and continue back. A spotter would also assist in tracking time to ensure participants completed the trial in the allotted time.

**(b) Treadmill Group**

Treadmill gait training required participants to walk on a treadmill presented with equally distributed standardized transverse white lines. All participants walked at the speed determined at pre-test. This speed was inputted by the student investigator prior to commencement of training.

A posttest was administered six weeks after the pretest, followed by a six week retention test. During the retention period, participants were told to exercise no more than usual.

### 2.4. Statistical Analysis

Long-term effects compared measurements across time placing pre-, post-, and retention test values in the same analysis of variance. The dependent variables analyzed were TUG times, 30-second chair stand, UPDRS III score, and all GAITRite measures. Step length and step time data were further analyzed according to more affected versus less affected lower limb. More affected lower limb was defined by summing left and right scores for question 27 and 28 of the UPDRS III (leg agility and leg tremor, resp.) and taking the greater score. However, after finding no differences, left and right limb data was automatically pooled by the statistical analysis software. Also, first and last walking trials of all GAITRite measures were taken out of the analysis to avoid any learning and fatigue effects. Analysis was carried out by STATISTICA 8.0 using a group (treadmill, overground, control) by time (pretest, posttest, and retention test) ANOVA. An alpha level of 0.05 was used in all analyses. A Tukey's honest significant difference (HSD) post hoc was further employed to determine from where the significant differences were driven.

## 3. Results

### 3.1. Baseline Comparisons

Baseline characteristics can be seen in Table 1. Although the OG group appears to be slightly older than TG and CG, one-way ANOVA's were conducted comparing all three groups for severity using the UPDRS Section 3 height, initial velocity, step length, and TUG times resulting in no significant differences (*P* = 0.81, *P* = 0.97, *P* = 0.32, *P* = 0.20, *P* = 0.16, resp.). 

### 3.2. Outcome Measures (For summary see Table 2)

**Figure 1:**
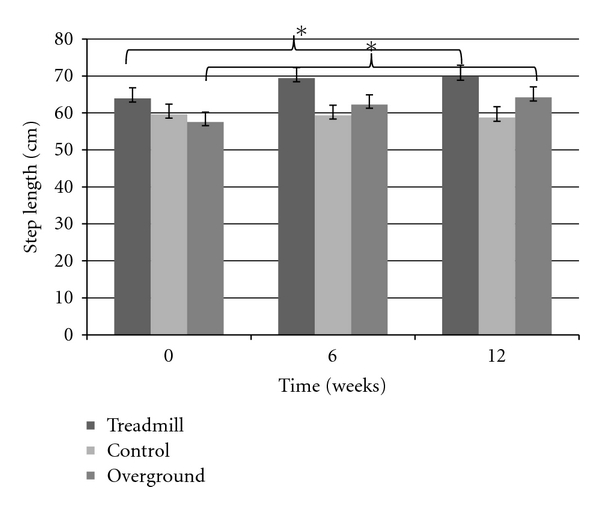
Step length significantly improves in TG and OG after six weeks (posttest) and is maintained after 12 weeks (retention test).

**Figure 2:**
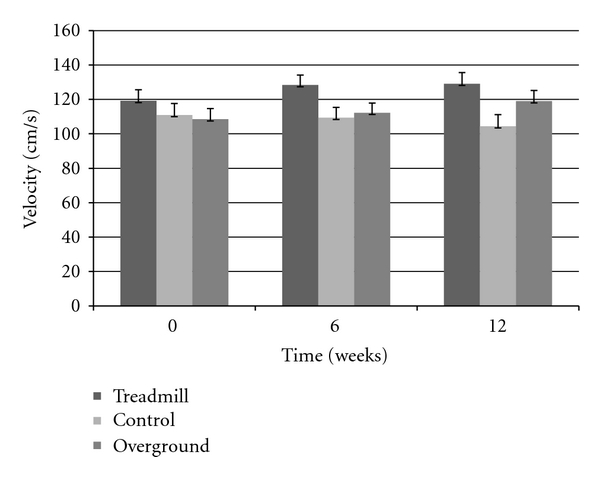
Velocity increases ~10 cm/s after 12-weeks (retention test) in only TG and OG.

**Figure 3:**
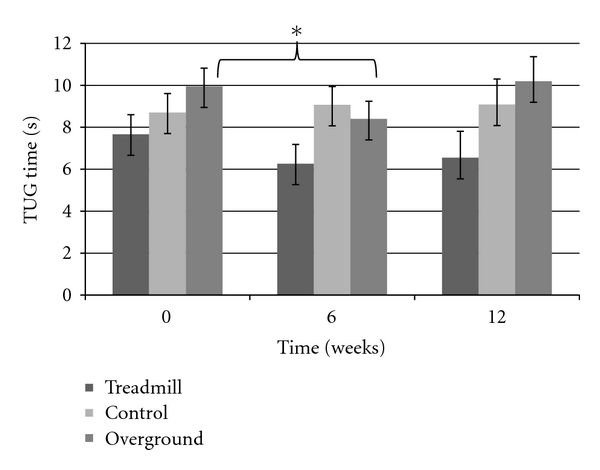
Examination of TUG times reveals a short-term main effect in PD OG.

**Figure 4:**
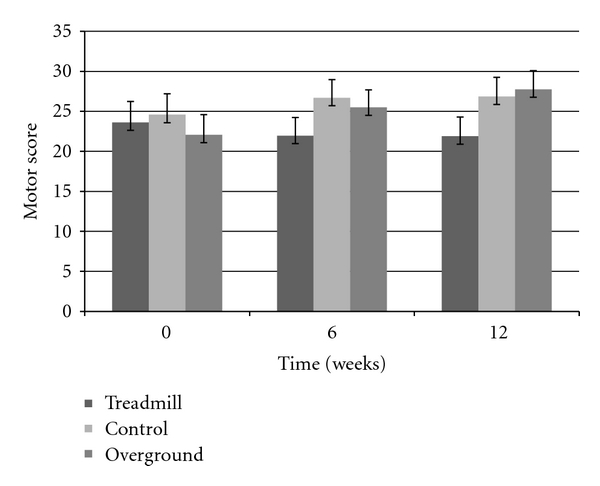
Motor scores showed at trend in improving symptoms in the TG, while the OG and CG seem to worsen symptoms.

Step length showed an overall group by time interaction (F_(4,72)_ = 4.5338, *P* < 0.003), post hoc analysis confirmed that both intervention group improved and maintained (after the retention period), whereas the control group showed no changes over time (Figure 1). Gait velocity also showed an overall group by time interaction (F_(4,72)_ = 3.7605, *P* < 0.008), with the interaction being driven by a velocity improvement in both training groups but not the control group. Figure 2 displays an overall ~10 cm/s increase in both TG and OG, while the CG decreased in gait speed; however, post hoc analysis revealed this change in velocity was not significant. There was no change seen in cadence and 30-second chair stand in all groups, across all three testing periods (*P* > 0.05). Examination of the TUG test revealed a significant group by time interaction (F_(2,39)_ = 4.0477, *P* < 0.05), suggesting that only the OG had decreased TUG times after the six week intervention. However, while still a significant interaction (F_(4,72)_ = 2.5564, *P* < 0.05), after three participants (two in TG, one in CG) were excluded from the analysis due to medical conditions at the retention period, improvements in TUG time for the OG returned to baseline values after time of retention (Figure 3). The UPDRS severity scores were analyzed and approached a significant interaction (*P* = 0.06) (Figure 4). The TG showed a trend to decrease symptom severity from pre- to posttest, and improvements were maintained over the retention period. Contrarily, the CG and OG showed a modest symptom severity increase (i.e., symptoms worsened) from pre- to posttest, which was also maintained after the retention test. All other spatial and timing gait parameters showed no change.

## 4. Discussion

While many studies have demonstrated the positive benefits associated with visually cued walking in PD, little to no studies have evaluated long term benefits of visually cued gait training. Here we present (according to “level of evidence” and “grading of evidence guidelines”) a Silver BIIa evidence study to evaluate the influence of long-term visual cue training. A primary objective of the current study was to isolate visual cues in a static versus dynamic context in order to understand the extent to which optic flow contributes to gait improvements in PD. The two gait interventions were conducted in nearly identical fashions, with the only difference between group training protocols being whether the cues were on the treadmill (TG) or on the ground (OG). In order to remove any other potential confounds, all other variables such as intensity, required step length, frequency of training, and duration of training were kept identical between groups. 

Parkinsonian gait has previously been theorized to be the result of a deficient connection between the basal ganglia and supplementary motor area (SMA). The interactions between these two structures are commonly associated with controlling well-learned movements. However, in PD, this disconnect is believed to cause impaired internal cueing within the basal ganglia, often manifesting itself into problematic walking. Visual cues are proposed to bypass this deficient loop and use visual motor pathways in the lateral premotor cortex (PMC) and posterior parietal cortex (PPC), as these areas are activated through externally cued movements and paradoxical movements, respectively [21]. Similar gait results in both training groups is evidence that usual optic flow is not essential, but rather, the transverse lines may be activating these areas regardless of surrounding environmental information.

It is important to acknowledge however, that we did not completely remove optic flow in the treadmill training group. Rather we were able to reduce the amount of optic flow available in the treadmill group relative to the overground group. Thus, some researchers might argue that as long as some optic flow is available, gait improvements can still be achieved. 

The findings of our study confirm that transverse lines have a positive impact on gait parameters [15, 17, 19, 22, 23] and contributes to the existing PD literature on the long-term effects of visual cue training protocols. Step length was shown to improve after six weeks and was maintained after an additional six-week retention period in both the TG and OG. Findings indicate that this spatial gait improvement is not the result of short-term training effects but rather a lasting change in the subjects walking. The fact that step length improvement was maintained even after the non-exercising period suggests that cueing provides potential for greater long-term retention gains [24]. The present study also rules out any potential strength gains that may have contributed to step length improvements. The 30-second chair stand was used as a tool to assess lower limb strength and gait performance [25] and showed no change across all groups. 

 A significant interaction revealed that both intervention groups achieved faster walking speeds upon completing the current study. Moreover, these same groups also experienced no change in cadence. In many cueing and treadmill studies, increase in step length is often accompanied by an increase in cadence [17, 26, 27]. However, it is unknown as to what extent each variable (step length or cadence) influences gait velocity. Our study reveals that PD individuals are achieving faster walking speeds due to taking larger steps rather than increasing the frequency of stepping. Thus, their velocity improvements were a result of step length gains rather than a compensatory reaction to cadence. 

Although walking measures were similar across the two intervention groups, the TUG test did reveal an important difference. The TUG test has been shown to significantly correlate with the Berg balance scale, implying that improvement in TUG times may suggest an improvement in dynamic balance as well [28]. Our study revealed that only the OG significantly improved TUG times. This may be due to the nature of the overground walking protocol, which required the participant to turn at the end of the visual cue carpet, mimicking the constant movement found in people walking on a treadmill. By having individuals turn at the end of the 16-meter carpet, the OG may have developed strategies for turning. The participants could have used the cues on the carpet to compete their turn, similar to the way they use transverse lines during straight line walking. The lines may have acted as a critical feedback tool for completing a successful turn, which is essential in optimizing motor performance [29]. This would suggest that this group may be simply using an attentional strategy in which the cues are used to focus ones attention on completing the required gait sequence [17]. Alternatively, it is possible that the OG had more opportunity to practise turns relative to the TG (since no turns are made on a treadmill). These results provide some important implications for rehabilitation professionals, as turning movements have been problematic in PD and closely linked to falling incidences and freezing episodes [30, 31]. These effects, however, did not persist past the posttest, which could be due to the complex nature of turning in general compared to straight walking. 

In assessing symptom severity, the UPDRS motor score displayed a trend towards a significant interaction (*P* = 0.06). More importantly, there is a hint of symptom improvement in the TG, while the OG and CG displayed worsening of symptoms that often accompanies the progression of the disease [32]. It is important to consider that the improvements in the TG may be caused by the treadmill belt driving proprioceptive inputs [33], when the lower limbs are actively and passively taken through the walking cycle. Impaired proprioception has been previously reported in PD individuals [34], and perhaps, the treadmill belt is externally stimulating the afferent inputs that may help overcome the secondary effects of the disease. Hence, future research might also consider how external drive may be related to treadmill training, while overground training might be more internally driven. Animal model studies looking at treadmill training have been shown to acutely increase dopamine release [35] and chronically upregulate D2 receptors in the striatum of rats [36]. The motor symptom improvement found in the TG may similarly be the result of this overall availability and utilization of dopamine.

## 5. Conclusions

The overall improvements found in the treadmill and overground groups as compared to the control group are indicative of the positive impact transverse lines have on gait. However, similar step length and velocity improvements in both training groups suggests that typical optic flow is not necessarily required to achieve short- and/or long-term benefits associated with PD gait training. Rather than using optic flow information, PD participants may be using vision as a strategy to overcome lower limb proprioceptive deficit and/or focus attention on consciously achieving the stepping process. Interestingly, hints of motor severity improvement in the TG seem to be driven by additional proprioceptive input fed by the belt, while functional tests such as the TUG improved for those that repetitively practised turning. The results of our study reveal that a reduced amount of optic flow can produce similar benefits during gait training, and clinically, the implementation of transverse lines as a long-term cueing therapy for Parkinson's disease seems appropriate. Furthermore, future work should focus on implementing visual cueing therapy during functional aspects of walking such as gait initiation, termination, and turning.

##  Author's Contributions

Each of the coauthors contributed equally to the development and completion of this project.

##  Conflict of Interests

The authors declare no potential conflict of interests with respect to the authorship and/or publication of this paper.

## Figures and Tables

**Table 1:** Characteristics of the three groups.

Group	Age-M (yrs)	Height-M (cm)	UPDRS-M (score)	Gender
PD TG	63.86 (8.41)	170.97 (10.29)	23.68 (10.1)	8 male, 6 female
PD OG	73.93 (6.53)	170.72 (10.22)	22.07 (8.0)	12 male, 2 female
PD CG	67.43 (9.26)	170.15 (6.83)	24.21 (9.5)	11 male, 3 female

*Note: *M denotes mean, standard deviations found in brackets.

**Table 2:** Mean (6 standard deviation) of outcome measures from pre-, post-, and retention test.

Measure	Test	PD control	PD treadmill group	PD overground group	ANOVA *Pre-, Post-, *and* Retention *
Step length (cm)	*Pretest*	57.7 (12.3)	63.9 (10.6)	57.6 (62.3)	*P* = 0.003
	*Posttest*	59.3 (12.7)	69.4 (9.9)**	62.3 (8.3)**	
	*Retention test*	58.8 (14.0)	69.9 (12.4)**	64.2 (10.3)**	

Velocity (cm/sec)	*Pretest*	109.0 (27.7)	119.2 (15.6)	108.5 (23.8)	*P* = 0.008
	*Posttest *	109.6 (27.1)	128.3 (16.5)	112.2 (18.1)	
	*Retention* *test *	104.3 (32.8)	129.1 (18.0)	118.9 (19.0)	

TUG time (seconds)	*Pretest*	9.0 (3.0)	7.7 (2.0)	9.9 (4.2)	*P* = 0.046
	*Posttest *	9.1 (3.3)	6.3 (2.0)	8.4 (3.7)	
	*Retention* *test *	9.1 (3.7)	6.5 (2.5)	10.2 (5.8)	

UPDRS score	*Pretest*	24.6 (9.7)	23.6 (10.5)	22.1 (8.0)	NS
	*Posttest *	26.7 (8.8)	23.0 (8.0)	25.5 (7.0)	
	*Retention* *test *	26.8 (8.8)	22.6 (8.0)	27.8 (9.1)	

NS: denotes a nonsignificant interaction.

**: denotes significantly different from pretest (*P* < .05).

3 patients removed from the current analysis.

## References

[1] Morris ME, Iansek R, Matyas TA, Summers JJ (1994). Ability to modulate walking cadence remains intact in Parkinson’s disease. *Journal of Neurology Neurosurgery and Psychiatry*.

[2] Glickstein M, Stein J (1991). Paradoxical movement in Parkinson’s disease. *Trends in Neurosciences*.

[3] Assaiante C, Marchand AR, Amblard B (1989). Discrete visual samples may control locomotor equilibrium and foot positioning in man. *Journal of Motor Behavior*.

[4] Gibson JJ (1954). The visual perception of objective motion and subjective movement. *Psychological Review*.

[5] Azulay JP, Mesure S, Amblard B, Blin O, Sangla I, Pouget J (1999). Visual control of locomotion in Parkinson’s disease. *Brain*.

[6] Prokop T, Schubert M, Berger W (1997). Visual influence on human locomotion. Modulation to changes in optic flow. *Experimental Brain Research*.

[7] Lamontagne A, Fung J, McFadyen BJ, Faubert J (2007). Modulation of walking speed by changing optic flow in persons with stroke. *Journal of NeuroEngineering and Rehabilitation*.

[8] Song JL, Hidler J (2008). Biomechanics of overground vs. treadmill walking in healthy individuals. *Journal of Applied Physiology*.

[9] Frenkel-Toledo S, Giladi N, Peretz C, Herman T, Gruendlinger L, Hausdorff JM (2005). Effect of gait speed on gait rhythmicity in Parkinson’s disease: variability of stride time and swing time respond differently. *Journal of NeuroEngineering and Rehabilitation*.

[10] Bello O, Sanchez JA, Fernandez-del-Olmo M (2008). Treadmill walking in Parkinson’s disease patients: adaptation and generalization effect. *Movement Disorders*.

[11] Suteerawattananon M, Morris GS, Etnyre BR, Jankovic J, Protas EJ (2004). Effects of visual and auditory cues on gait in individuals with Parkinson’s disease. *Journal of the Neurological Sciences*.

[12] Lebold CA, Almeida QJ (2011). An evaluation of mechanisms underlying the influence of step cues on gait in Parkinson's disease. *Journal of Clinical Neuroscience*.

[13] Lebold CA, Almeida QJ (2010). Evaluating the contributions of dynamic flow to freezing of gait in parkinson’s disease. *Parkinson’s Disease*.

[14] Nieuwboer A (2008). Cueing for freezing of gait in patients with Parkinson’s disease: a rehabilitation perspective. *Movement Disorders*.

[15] Purdon Martin J (1977). The basal ganglia and postural mechanisms. *Agressologie*.

[16] Jiang Y, Norman KE (2006). Effects of visual and auditory cues on gait initiation in people with Parkinson’s disease. *Clinical Rehabilitation*.

[17] Morris ME, Iansek R, Matyas TA, Summers JJ (1996). Stride length regulation in Parkinson’s disease: normalization strategies and underlying mechanisms. *Brain*.

[18] van Wegen E, Lim I, De Goede C (2006). The effects of visual rhythms and optic flow on stride patterns of patients with Parkinson’s disease. *Parkinsonism and Related Disorders*.

[19] Sidaway B, Anderson J, Danielson G, Martin L, Smith G (2006). Effects of long-term gait training using visual cues in an individual with Parkinson disease. *Physical Therapy*.

[20] Podsiadlo D, Richardson S (1991). The timed “Up and Go”: a test of basic functional mobility for frail elderly persons. *Journal of the American Geriatrics Society*.

[21] Hanakawa T, Fukuyama H, Katsumi Y, Honda M, Shibasaki H (1999). Enhanced lateral premotor activity during paradoxical gait in parkinson’s disease. *Annals of Neurology*.

[22] Marchese R (2000). The role of sensory cues in the rehabilitation of Parkinsonian patients: a comparison of two physical therapy protocols. *Movement Disorders*.

[23] Ferrarin M, Rabuffetti M, Tettamanti M, Pignatti R, Mauro A, Albani G (2008). Effect of optical flow versus attentional strategy on gait in Parkinson’s disease: a study with a portable optical stimulating device. *Journal of NeuroEngineering and Rehabilitation*.

[24] Platz T, Brown RG, Marsden CD (1998). Training improves the speed of aimed movements in Parkinson’s disease. *Brain*.

[25] Masuda Y, Nisida Y, Kurosawa K (2004). Relationship of a 30-second chair-stand test to gait performance in stroke patients. *Rigakuryoho Kagaku*.

[26] Lindquist AR, Prado CL, Barros RML, Mattioli R, Lobo Da Costa PH, Salvini TF (2007). Gait training combining partial body-weight support, a treadmill, and functional electrical stimulation: effects on poststroke gait. *Physical Therapy*.

[27] Toole T, Maitland CG, Warren E, Hubmann MF, Panton L (2005). The effects of loading and unloading treadmill walking on balance, gait, fall risk, and daily function in Parkinsonism. *NeuroRehabilitation*.

[28] Bennie S (2003). Measurements of balance: comparison of the timed “Up and Go” test and functional reach test with the berg balance scale. *Journal of Physical Therapy Science*.

[29] Carr J, Shepard RB (1998). *Neurological Rehabilitation: Optimizing Motor Performance*.

[30] Stack E, Ashburn A (1999). Fall events described by people with Parkinson’s disease: implications for clinical interviewing and the research agenda. *Physiotherapy Research International*.

[31] Rahman S, Griffin HJ, Quinn NP, Jahanshahi M (2008). The factors that induce or overcome freezing of gait in Parkinson’s disease. *Behavioural Neurology*.

[32] Alves G, Wentzel-Larsen T, Aarsland D, Larsen JP (2005). Progression of motor impairment and disability in Parkinson disease: a population-based study. *Neurology*.

[33] Bello O, Marquez G, Camblor M, Fernandez-Del-Olmo M (2010). Mechanisms involved in treadmill walking improvements in Parkinson’s disease. *Gait and Posture*.

[34] Abbruzzese G, Berardelli A (2003). Sensorimotor integration in movement disorders. *Movement Disorders*.

[35] Meeusen R, Piacentini MF, De Meirleir K (2001). Brain microdialysis in exercise research. *Sports Medicine*.

[36] MacRae PG, Spirduso WW, Walters TJ (1987). Endurance training effects on striatal D2 dopamine receptor binding and striatal dopamine metabolites in presenescent older rats. *Psychopharmacology*.

